# Autophagy-Related Proteins in Triple-Negative Breast Cancer: From Molecular Insights to Therapeutic Applications

**DOI:** 10.3390/ijms26189231

**Published:** 2025-09-21

**Authors:** Meng-Ke Ma, Da-Qiang Li

**Affiliations:** 1 Department of Oncology, Cancer Institute, Shanghai Medical College, Fudan University, Shanghai 200032, China; cococlera@163.com; 2 Shanghai Key Laboratory of Breast Cancer, Department of Breast Surgery, Fudan University Shanghai Cancer Center, Shanghai 200032, China

**Keywords:** autophagy, triple-negative breast cancer, cancer progression, therapeutic responsiveness

## Abstract

Triple-negative breast cancer (TNBC) represents the most aggressive and therapeutically recalcitrant breast cancer subtype, exhibiting dismal clinical outcomes due to its intrinsic heterogeneity and lack of molecularly targeted treatment options. Emerging evidence has established the autophagy-related proteins (ARPs) as key regulators of TNBC pathogenesis, functioning not only as metabolic gatekeepers but also as multifaceted modulators of malignant transformation, disease progression, and therapeutic responsiveness. These proteins exert diverse functions in TNBC through both canonical autophagy-dependent pathways and non-canonical mechanisms. In this review, we critically examine the pleiotropic functions and molecular mechanisms of ARPs in TNBC progression and therapeutic responsiveness, with special emphasis on their context-dependent roles in both fortifying therapeutic resistance and, paradoxically, creating vulnerabilities for therapeutic exploitation.

## 1. Introduction

Triple-negative breast cancer (TNBC) is a clinically aggressive and molecularly heterogeneous malignancy, which is characterized by a pronounced tendency for early recurrence and distant metastasis as well as significantly worse survival outcomes compared to other breast cancer subtypes [1]. Owing to the lack of estrogen receptor (ER), progesterone receptor (PR), and human epidermal growth factor receptor 2 (HER2) expression, TNBC is devoid of established targeted therapies, rendering systemic chemotherapy the mainstay of treatment [2]. These clinical challenges underscore the pressing need to elucidate novel molecular vulnerabilities in TNBC pathogenesis, develop biomarker-guided therapeutic strategies, and advance precision oncology frameworks to improve survival outcomes for TNBC patients.

Autophagy is an evolutionarily conserved lysosomal degradation process that maintains cellular homeostasis by selectively eliminating damaged organelles and misfolded proteins [3,4]. Accumulating evidence shows that autophagy plays pivotal roles in malignant transformation, tumor evolution, and therapeutic resistance in human cancers [4,5]. In particular, the functional consequences of autophagy in human cancers exhibit remarkable context-dependence [4,5]. In this case, autophagy acts as a tumor-suppressive mechanism during initial stage of tumorigenesis through maintaining genomic integrity and mitigating chronic inflammatory responses. In advanced malignancies, however, autophagy is co-opted by cancer cells to promote cancer progression, metastatic dissemination, and therapeutic resistance by sustaining energy homeostasis and supplying critical metabolic precursors [6,7,8].

Notably, TNBC often exhibits elevated autophagic flux, which functions as a critical survival strategy that enables cancer cells to withstand metabolic stress and thrive in the nutrient-deprived tumor microenvironment [9]. Aberrant expression of autophagy-related proteins (ARPs) in TNBC drives cancer progression and confers therapeutic resistance, underscoring their potential as promising molecular targets for cancer therapy [10,11]. While accumulating evidence has established ARPs as crucial players in TNBC pathogenesis, their clinical translation remains challenging. The pleiotropic effects and context-dependent regulation of autophagy create substantial hurdles for targeted therapy, compounded by our incomplete understanding of the intricate molecular networks involved. Addressing these challenges will be essential for developing effective precision therapies that harness autophagy modulation to enhance TNBC therapy efficacy.

In this review, we systematically integrate current knowledge on ARPs in TNBC, elucidating their multifaceted roles in tumor initiation, progression, and therapeutic resistance. By critically evaluating the underlying molecular mechanisms, we provide a comprehensive assessment of their dual potential as both actionable therapeutic targets and predictive biomarkers for clinical application.

## 2. Molecular Regulatory Network of Autophagy

Generally, autophagy is mechanistically categorized into three main types, including macroautophagy, microautophagy, and chaperone-mediated autophagy (CMA) [3,4]. Of them, macroautophagy (hereafter referred to as autophagy) is the most extensively characterized type and involves autophagosome formation and subsequent lysosomal fusion [3,4]. Microautophagy is characterized by the direct sequestration of cytoplasmic cargo through invagination of the lysosomal membrane [12]. In contrast, CMA specifically recognizes cytosolic substrates containing a pentapeptide KFERQ motif (or its biochemically similar variants), which are recognized by the molecular chaperone heat shock protein family A member 8 (HSPA8). This chaperone–substrate complex then docks at lysosomal-associated membrane protein 2A (LAMP-2A) receptor, where the target protein is unfolded and translocated across the lysosomal membrane for degradation [13]. These autophagic pathways are tightly regulated by a complex network of ARPs.

**Figure 1:**
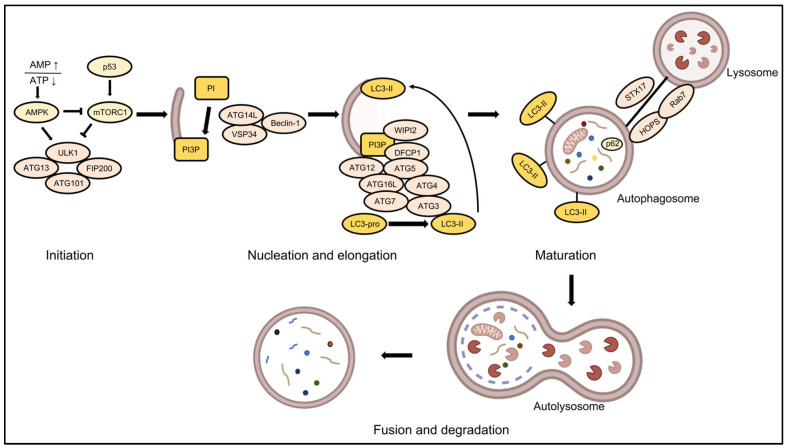
Molecular regulatory network of autophagy cascade. Autophagy is triggered by diverse stress signals via a tightly regulated molecular cascade. The process initiates with the activation of the class III PI3K complex and the recruitment of autophagy-related proteins (ARPs), which coordinately drive phagophore nucleation and membrane expansion. During autophagosome maturation, selective incorporation of membrane components and cargo receptors occurs through highly specific molecular sorting mechanisms. Ultimately, mature autophagosomes fuse with lysosomes to form autolysosomes, where sequestered cargo is degraded and metabolic byproducts are recycled to sustain cellular homeostasis. This image was created with BioRender.com (accessed on 20 July 2025) AMPK, AMP-activated protein kinase; mTORC1, mechanistic target of rapamycin complex 1; ULK1, unc-51-like kinase 1; FIP200, family interacting protein of 200 kDa; PI, phosphatidylinositol; PI3P, phosphatidylinositol 3-phosphate; VSP34, vacuolar protein sorting 34; WIPI2, WD repeat domain phosphoinositide-interacting protein 2; LC3, microtubule-associated protein 1 light chain 3; DFCP1, double FYVE-containing protein 1; STX17, Syntaxin 17; HOPS, homotypic fusion and vacuole protein sorting complex; Rab7, ras-related protein Rab-7a. Upward arrows (↑) denote activation or increased levels, while downward arrows (↓) denote inhibition or decreased levels.

The autophagic cascade occurs through a series of sequential and tightly regulated stages, including initiation via autophagy-related protein kinase complexes, phagophore nucleation and membrane elongation, culminating in autophagosome formation, autophagosome–lysosome fusion generating degradative autolysosomes, and subsequent cargo degradation in lysosomes [3,4,14,15,16] (Figure 1).

Autophagy initiation is principally governed by the unc-51-like autophagy-activating kinase 1 (ULK1) complex, which integrates opposing signals from the mechanistic target of rapamycin (mTOR) and AMP-activated protein kinase (AMPK) pathways [16,17,18]. Under nutrient-rich conditions, mTOR complex 1 (mTORC1) actively suppresses autophagy by phosphorylating ULK1 at Ser757, disrupting its interaction with AMPK and maintaining the complex in an inactive state [17,19]. Conversely, energy deprivation triggers AMPK-mediated phosphorylation of ULK1 at distinct residues (Ser317/Ser777), promoting ULK1 complex assembly and autophagosome biogenesis [17].

The nucleation of autophagosomes is governed by the class III phosphatidylinositol 3-kinase (PI3K) complex, which consists of the catalytic subunit VPS34, the scaffold protein Beclin-1, the autophagy-specific adaptor ATG14L, and additional regulatory subunits [20]. This macromolecular assembly generates phosphatidylinositol 3-phosphate (PI3P) as a critical lipid second messenger, which subsequently recruits PI3P-binding effector proteins to orchestrate phagophore membrane elongation and curvature formation [9,21,22]. Autophagosome membrane elongation is facilitated by two interdependent processes, including covalent conjugation of the ATG12-ATG5-ATG16L1 complex to the phagophore membrane and phosphatidylethanolamine (PE)-mediated lipidation of LC3 to form LC3-II. The lipidated LC3-II becomes stably associated with autophagosomal membranes, serving as both a structural component essential for autophagosome maturation and a canonical biomarker for monitoring autophagic flux [23,24].

The final step in autophagy involves selective cargo recognition via LC3-II-interacting receptors, SNARE (soluble NSF attachment protein receptor)-mediated fusion of the autophagosome with the lysosome (primarily through STX17-SNAP29-VAMP8 complexes), and formation of degradative autolysosomes where lysosomal hydrolases process sequestered cargo for macromolecular recycling [20,25,26,27].

Notably, beyond their canonical functions in autophagy regulations, many ARPs exhibit pleiotropic functions through participation in diverse cellular signaling pathways independent of autophagy [28,29]. These moonlighting activities of ARPs significantly influence oncogenic signaling networks and therapeutic resistance pathways [30].

## 3. Context-Dependent Roles of ARPs in TNBC Progression

**Figure 2:**
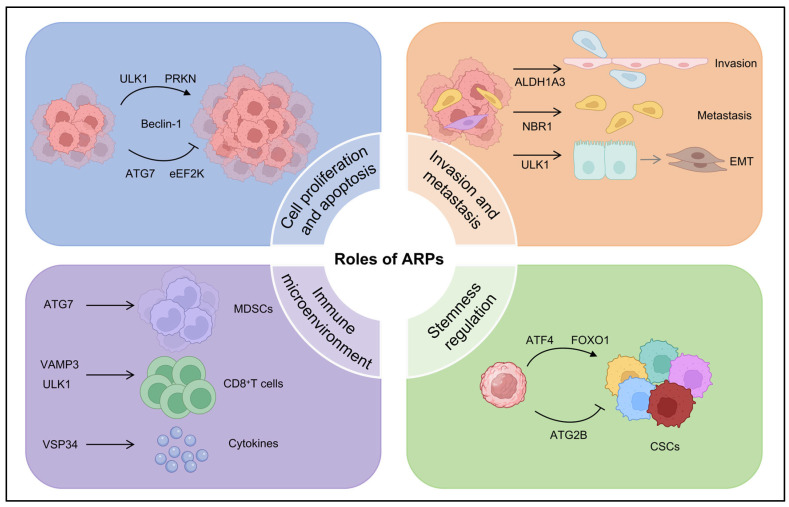
Roles of ARPs in TNBC progression. In TNBC, ARPs drive tumor progression by regulating proliferation and apoptosis, enhancing invasion and metastasis (via EMT), remodeling the immune microenvironment (affecting immune cells and cytokines), and sustaining cancer stem cell stemness. This image was created with BioRender.com (accessed on 23 July 2025).

Emerging evidence reveals that ARPs exert diverse functions in TNBC through both canonical autophagy-dependent pathways and non-canonical mechanisms. These multifaceted roles include metabolic reprogramming to fulfill biosynthetic demands and promote stress adaptation, potentiation of invasive potential and metastatic spread, dynamic modulation of the tumor microenvironment, and maintenance of cancer stem cell properties. Through these coordinated actions, ARPs drive TNBC pathogenesis at multiple levels from sustaining cellular homeostasis to accelerating tumor progression and therapeutic resistance (Figure 2).

### 3.1. Regulation of Cell Proliferation and Apoptosis by ARPs in TNBC

ARPs serve as multifunctional regulators that integrate basal metabolic homeostasis with oncogenic signaling networks in TNBC. Through dynamic crosstalk with cell-cycle checkpoints, apoptotic machinery, and metabolic sensors, these proteins establish sophisticated control circuits governing cancer cell proliferation and survival.

It has been documented that Beclin-1, a core component of the class III phosphatidylinositol 3-kinase complex essential for autophagy initiation [31], is overexpressed in most TNBC cell lines and clinical samples, and strongly correlates with enhanced proliferation [32,33]. Conversely, genetic depletion of Beclin-1 induces cell-cycle arrest at G0/G1 phase, leading to significant suppression of TNBC proliferation [33]. Paradoxically, pharmacological activation of Beclin-1-mediated autophagy potently inhibits TNBC cell growth [34]. This functional dichotomy underscores context-dependent roles of Beclin-1 in TNBC pathogenesis, where its ultimate biological effects are determined by microenvironmental cues and the dynamic regulation of autophagic flux.

The mTOR pathway serves as a master regulator that coordinately suppresses autophagy while promoting cell proliferation and cell-cycle progression [35]. Under nutrient-replete conditions, mTORC1 phosphorylates and inactivates the ULK1 kinase complex, effectively suppressing autophagosome initiation [17]. Emerging studies reveal that mTOR-driven TNBC progression involves sophisticated crosstalk with autophagy machinery, forming reciprocal regulatory circuits that maintain a proliferation-permissive tumor microenvironment [36]. Pathological hyperactivation of mTOR signaling in TNBC induces dual oncogenic effects, including metabolic reprogramming through aberrant nutrient sensing and loss of cell-cycle control via dysregulated cyclin-CDK activity [35,37,38].

ARPs also play a pivotal role in modulating the apoptotic threshold of TNBC cells through dual regulatory mechanisms. For instance, it has been shown that ATG7, a core autophagy-related gene encoding protein that is indispensable to classical degradative autophagy [39], exerts a pro-apoptotic function by activating the caspase-3/PARP cleavage cascade [40]. However, genetic depletion of ATG7 (or Beclin-1) in TNBC cells induces apoptosis by inactivating STAT3 signaling [41]. These findings reveal an intricate balance wherein ARPs can either promote or suppress apoptosis depending on cellular context and pathway activation status.

Collectively, these findings position selective ARPs as clinically actionable targets for TNBC therapy. By precisely modulating key nodes in autophagic networks, particularly those governing proliferative signaling and stress adaptation, we may develop innovative treatment strategies to disrupt tumor survival mechanisms while overcoming therapeutic resistance. This approach holds significant promise for addressing the critical unmet need in TNBC management, where current therapeutic options remain limited.

### 3.2. Regulation of Invasive and Metastatic Potential of TNBC Cells by ARPs

TNBC exhibits enhanced invasive and metastatic potential with significantly higher rates of distant metastasis at diagnosis than other breast cancer subtypes [1,42]. Emerging evidence reveals that autophagy contributes substantially to this malignant phenotype through multifaceted mechanisms, including facilitating epithelial–mesenchymal transition (EMT) via transcriptional reprogramming, enabling cytoskeletal reorganization through Rho GTPase modulation, and activating pro-metastatic signaling pathways, including transforming growth factor-β (TGF-β) and Wnt/β-catenin cascades [4,43,44,45]. These autophagy-mediated processes collectively enhance cancer cell motility, intravasation, and survival during metastatic colonization.

The serine/threonine kinase ULK1, while best characterized for its role in autophagy initiation [16,17,18], has been shown to regulate TNBC metastasis through both autophagic and non-autophagic mechanisms. In non-TNBC breast cancers, ULK1 functions as a metastasis suppressor by binding to Paxillin, a key mechanotransduction adaptor protein. This interaction disrupts actin stress fiber polymerization and focal adhesion complex assembly, ultimately impairing cellular contractility and motility [46]. Paradoxically, in TNBC models, ULK1 overexpression drives metastatic progression via two distinct mechanisms, including autophagy-dependent upregulation of EMT transcription factors (e.g., Snail) and extracellular matrix components (e.g., fibronectin), and hypoxia-mediated remodeling of the tumor microenvironment [47,48]. Intriguingly, ULK1 inhibition under hypoxia induces aberrant fibronectin deposition, which activates integrin/FAK/Src signaling cascades and fosters a pro-metastatic niche that enhances pulmonary colonization [47,48]. These findings underscore the context-dependent duality of ULK1 in TNBC metastasis.

The molecular chaperone HSPA8, a critical regulator of selective autophagy [13], demonstrates significant overexpression in TNBC and exhibits context-dependent modulation of metastatic progression [49]. Mechanistically, HSPA8 displays dual and seemingly opposing functions. It potentiates metastatic capacity by activating Wnt/β-catenin signaling to enhance cancer cell motility [50], while concurrently restricting TNBC metastasis through lysosome-mediated degradation of transcription factor Snail [51]. In TNBC preclinical models, targeting HSPA8 exerts potent anti-metastatic effects by sustaining ERK pathway activation and disrupting β-catenin-mediated cell adhesion, collectively impairing migration and invasion [52]. These findings position HSPA8 as a promising molecular target for intercepting TNBC metastasis, though its therapeutic exploitation will require careful consideration of its pleiotropic functions.

**Figure 3:**
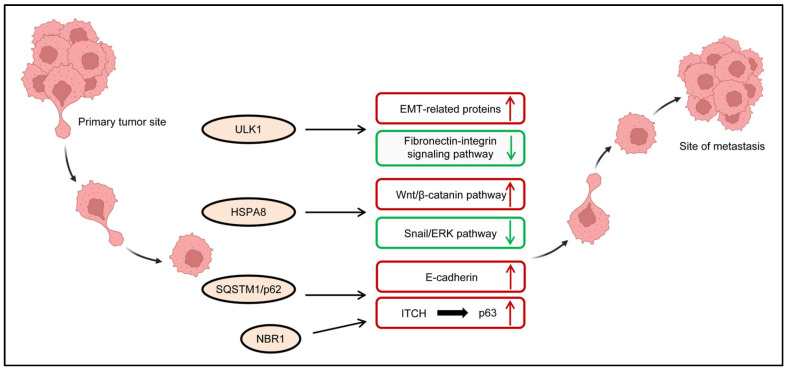
The Role of ARPs in TNBC Invasion and Metastasis. ARPs play critical roles in modulating tumor invasion and metastasis in TNBC. Red boxes with upward arrows denote factors or pathways that promote TNBC metastasis, whereas green boxes with downward arrows represent those that suppress it. This image was created with BioRender.com (accessed on 25 July 2025).

The autophagy-metastasis network in TNBC involves multiple additional regulatory components that orchestrate distinct aspects of metastatic progression (Figure 3). The selective autophagy receptor NBR1 (next to BRCA1 gene 1 protein) cooperates with SQSTM1/p62 to sequester ITCH, an E3 ubiquitin ligase that targets p63 for proteasomal degradation in breast cancer cells. This NBR1-mediated ITCH sequestration stabilizes p63 protein levels, thereby reinforcing the aggressive basal-like phenotype characteristic of metastatic TNBC [53]. SQSTM1/p62 enhances metastatic potential by directly binding E-cadherin and promoting its lysosomal degradation, thereby compromising adherens junction stability and increasing cellular invasiveness [54]. These findings collectively reveal how diverse autophagy components converge to promote TNBC metastasis through both degradative and non-degradative mechanisms.

### 3.3. Regulation of Cancer Cell Stemness by ARPs in TNBC

Cancer stem cells (CSCs) constitute a critical cellular subpopulation that drives therapeutic resistance, disease recurrence, and metastatic progression in TNBC [55]. Accumulating evidence reveals that ARPs function as essential regulators of CSC maintenance through multiple mechanisms, including preserving stemness properties via selective organelle and protein turnover, mitigating oxidative and metabolic stress to sustain the quiescent CSC state, and activating core stemness signaling pathways through non-degradative functions [56]. By orchestrating these complementary processes, ARPs establish a protective niche that reinforces TNBC CSC resilience, ultimately fostering cancer aggressiveness and therapy refractoriness.

Recent studies reveal that multiple ARPs exert distinct effects on CSC properties in TNBC through diverse molecular mechanisms. For instance, autophagy-related protein 2 homolog B (ATG2B) serves as a critical regulator of autophagic flux that constrains CSC properties in TNBC through its essential role in autophagosome biogenesis and maturation [57]. Similarly, the tumor suppressor DAB2IP (disabled homolog 2-interacting protein) constrains CSC maintenance in TNBC through autophagy-dependent mechanisms [58]. DAB2IP loss leads to SOX2/Nanog upregulation and enhanced self-renewal, coinciding with impaired autophagic flux [58]. Under basal conditions, the oncogenic aurora kinase A (AURKA) undergoes autophagic–lysosomal degradation. However, when phosphorylated at Thr288 by eukaryotic elongation factor 2 kinase (eEF2K), AURKA becomes resistant to autophagic degradation while simultaneously upregulating transcription factor SOX-8 expression. This creates a feed-forward loop that promotes proliferation, enhances invasive capacity, and maintains CSC properties in TNBC [59]. These findings demonstrate how oncogenic signaling networks interface with autophagy pathways to coordinate malignant progression and stem cell plasticity in TNBC.

Collectively, these findings demonstrate how oncogenic signaling networks interface with autophagy pathways to coordinate malignant progression and stem cell plasticity in TNBC. These coordinated actions establish an integrated “autophagy–stemness” axis that critically governs CSC self-renewal, differentiation plasticity, and therapeutic resistance. Targeted disruption of this network, either by selectively inhibiting pro-stemness autophagy factors or inducing the degradation of stemness markers, offers a promising therapeutic strategy to deplete the CSC pool, reduce tumor aggressiveness, and overcome therapeutic resistance in TNBC. This approach may yield novel combinatorial therapies that simultaneously target bulk tumor cells and the CSC niche.

### 3.4. Regulation of Tumor Immune Microenvironment by ARPs in TNBC

The tumor immune microenvironment constitutes a critical determinant of TNBC progression and therapeutic responsiveness [60,61]. Within this dynamic ecosystem, cancer cells actively orchestrate immune cell recruitment, cytokine networks, and antigen presentation machinery. Emerging evidence positions ARPs as pivotal regulators of tumor immune microenvironment remodeling, functioning as molecular interfaces that integrate cancer cell metabolism with immunomodulation. This regulation manifests as a dual-edged sword. While selective autophagy can promote antigen presentation and T-cell infiltration, sustained autophagic flux may alternatively foster immunosuppressive niches through PD-L1 (programmed cell death 1 ligand 1) stabilization and regulatory T-cell expansion.

Accumulating evidence shows that ARPs exert a complex dual regulatory role in modulating the immune microenvironment of TNBC. In this context, glycolytic restriction triggers ULK1-dependent autophagic degradation of liver-enriched activator protein (LAP), thus suppressing myeloid-derived suppressor cell (MDSC) recruitment to enhance CD8^+^ T cell function and inhibit TNBC progression [62]. Conversely, genetic ablation of ATG7 not only suppresses autophagic flux but also induces MDSC recruitment, thereby counteracting ULK1-mediated immunosuppression alleviation to reinforce an immune-suppressive tumor microenvironment [63,64]. Similarly, autophagy modulation demonstrates bidirectional effects on T cell functionality. Impaired autophagy in TNBC cells compromises T cell clonal expansion and activation, ultimately attenuating cytotoxic responses and promoting immune escape [65]. Paradoxically, hyperactivation of autophagy by vesicle-associated membrane protein 3 (VAMP3) promotes PD-L1^+^ exosome secretion, leading to PD-1-dependent CD8^+^ T cell suppression and immune evasion [66,67].

Beyond its established role in adaptive immunity, emerging evidence reveals that ARPs critically modulate innate immune signaling cascades. Pharmacological inhibition or genetic depletion of the class III phosphoinositide 3-kinase vacuolar protein sorting 34 (VPS34) triggers a robust activation of the STING-dependent DNA sensing pathway, inducing innate immune activation through natural killer (NK) cell and tumor-associated macrophage (TAM) recruitment to create an immunogenic tumor microenvironment [68].

In summary, ARPs orchestrate complex, bidirectional immunomodulatory effects within the TNBC microenvironment through multiple interconnected mechanisms. These proteins function as molecular rheostats that can either potentiate or suppress antitumor immunity depending on specific autophagic substrates degraded, cellular stress contexts, and immune cell populations involved. This complex regulation highlights the need for precision autophagy modulation in immunotherapy, requiring dual assessment of tumor autophagic activity and immune contexture to maximize efficacy while preventing immunosuppressive side effects.

## 4. Regulation of Therapeutic Responsiveness by ARPs in TNBC

**Figure 4:**
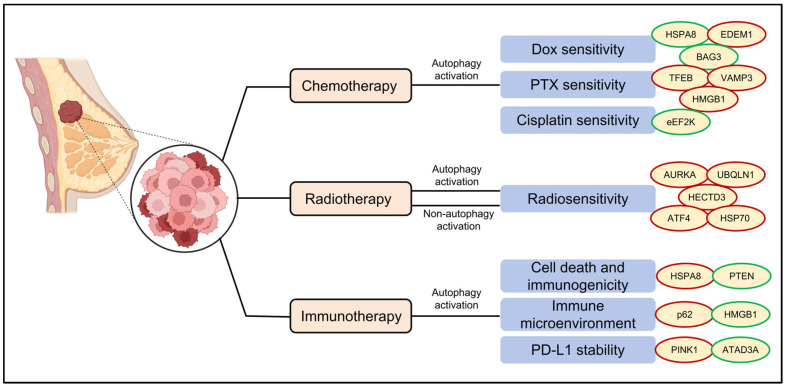
The role of ARPs in therapeutic responsiveness in TNBC. ARPs critically regulate TNBC treatment responses to chemotherapy, radiotherapy, and immunotherapy. Distinct ARPs mediate each of these therapeutic response pathways. They exert both autophagy-dependent and -independent effects (particularly in radiotherapy). Proteins highlighted in red indicate promotion of therapy resistance, whereas proteins highlighted in green indicate inhibition of resistance or enhancement of therapy sensitivity. This image was created with BioRender.com (accessed on 2 August 2025). DOX, doxorubicin; PTX, paclitaxel. PD-L1, programmed cell death 1 ligand 1.

ARPs serve as critical determinants of therapeutic responsiveness in TNBC, exhibiting dual roles in therapeutic resistance and sensitivity. On one hand, they facilitate chemoresistance and radioresistance by activating protective autophagy to eliminate therapy-induced cytotoxic damage and promote cellular repair. On the other hand, these proteins modulate immunotherapy efficacy through two key mechanisms, including direct regulation of immune checkpoint molecules (e.g., PD-L1 trafficking and degradation) and dynamic remodeling of the tumor immune microenvironment. A comprehensive understanding of these context-dependent mechanisms is essential for developing precision treatment strategies that either inhibit or harness autophagy to overcome therapeutic resistance in TNBC (Figure 4).

### 4.1. Chemotherapy

Chemotherapy continues to serve as the foundation of systemic therapy for TNBC, particularly in neoadjuvant and metastatic treatment paradigms [69,70,71]. Nevertheless, both intrinsic and acquired resistance mechanisms frequently compromise therapeutic efficacy. Emerging evidence has established that ARPs critically regulate TNBC chemosensitivity through multifaceted biological processes, including drug efflux, DNA damage repair, and apoptosis evasion [9].

Anthracyclines, particularly doxorubicin (DOX), remain cornerstone chemotherapeutics for TNBC, with their clinical effectiveness significantly modulated by autophagy regulation [72]. Recent studies reveal a dual role of autophagy in modulating DOX sensitivity. The triterpenoid pristimerin sensitizes TNBC cells to DOX by promoting HSPA8 ubiquitination and subsequent proteasomal degradation [52]. Conversely, endoplasmic reticulum degradation-enhancing alpha-mannosidase-like protein 1 (EDEM1) confers DOX resistance in TNBC cells partially through autophagy suppression [73]. Moreover, preclinical data demonstrate that pharmacologic autophagy blockade synergizes with conventional chemotherapy agents. In clinically relevant patient-derived xenograft (PDX) models of TNBC, combining autophagy inhibitor chloroquine with the first-line DOX/cyclophosphamide chemotherapy drugs significantly enhances tumor suppression and limits metastasis compared to monotherapy [74,75,76]. Bcl-2–associated athanogene 3 (BAG3) inhibition depletes autophagic LC3-II levels and overcomes dual resistance to DOX and 5-fluorouracil in treatment-refractory TNBC models [77]. Collectively, these results position autophagy modulation as a promising therapeutic approach to reverse anthracycline resistance in TNBC.

In addition, resistance to microtubule-targeting agents, such as paclitaxel (PTX), continues to pose a significant clinical challenge in TNBC treatment [78]. Emerging evidence highlights autophagy as a critical regulator of this resistance, with multiple mechanisms contributing to decreased drug sensitivity. Zhang et al. [79] demonstrated that E3 ubiquitin/ISG15 ligase TRIM25 catalyzes K63-linked polyubiquitination of transcription factor EB (TFEB). This post-translational modification promotes TFEB nuclear accumulation and subsequent activation of autophagy-related gene networks, ultimately diminishing PTX sensitivity in TNBC models. Similarly, overexpression of the SNARE protein VAMP3 sustains autophagic activity, enabling tumor cells to evade PTX-induced cytotoxicity [66]. Conversely, Tanshinone I enhances the sensitivity of TNBC cells to PTX by suppressing late-phase autophagy suppresses autophagy [80]. Inhibition of high-mobility group protein B1 (HMGB1), a key regulator of autophagy [81], sensitizes resistant TNBC cells to PTX by disrupting autophagosome–lysosome fusion [82]. These findings collectively establish aberrant autophagy activation as a key driver of PTX resistance in TNBC, while targeted autophagy inhibition presents a promising strategy to overcome therapeutic limitations.

Beyond anthracyclines and microtubule-targeting agents, autophagy critically regulates TNBC chemosensitivity to other agents. For instance, eEF2K inhibition activates AMPK-mediated autophagy, dramatically increasing cisplatin sensitivity in TNBC cells [83].

Emerging evidence reveals that non-coding RNAs orchestrate chemotherapy responses in TNBC through autophagy regulatory networks. For instance, long noncoding RNA (lncRNA) DDIT4-AS1 increases paclitaxel sensitivity by inhibiting mTOR signaling to downregulate autophagy effectors, including LC3-II and ATG5 [84]. This mechanistic insight exemplifies the broader paradigm wherein ARPs and their upstream regulators, including microRNAs (miRNAs), lncRNAs, circular RNAs (circRNAs), serve as critical determinants of chemotherapeutic efficacy [85]. The multifaceted involvement of these molecules in therapeutic resistance highlights their potential as both predictive biomarkers and therapeutic targets for overcoming chemoresistance in TNBC.

### 4.2. Radiotherapy

Radiotherapy (RT) serves as a cornerstone treatment for local tumor control and multimodal therapy in TNBC. The primary cytotoxic effects of RT are mediated through three key mechanisms, including induction of DNA double-strand breaks (DSBs), generation of reactive oxygen species (ROS), and initiation of lipid peroxidation cascades, which collectively induce irreversible tumor cell death [86,87,88].

TNBC cells often acquire radioresistance by activating protective autophagy pathways, which provide survival advantages during RT [30,89]. For instance, E3 ubiquitin-protein ligase HECTD3 promotes radioresistance of TNBC cell survival by enhancing autophagy [90]. Conversely, MiR-200c inhibits irradiation-induced autophagy and sensitizes TNBC cells to irradiation [91]. Therapeutic targeting of irradiation-induced autophagy, particularly through pharmacological blockade of checkpoint kinase 1 (Chk1), disrupts cytoprotective autophagy, exacerbating radiation-induced DNA damage and enhancing tumor cell killing [92]. The lysosomotropic compound chloroquine exerts dual anti-tumor effects by blocking autophagic flux through lysosomal inhibition while suppressing pro-metastatic cytokines, thereby simultaneously enhancing radiosensitivity and inhibiting radiation-induced metastasis in TNBC [93]. Collectively, these discoveries establish autophagy activation as a fundamental adaptive response that enables TNBC cells to resist radiotherapy.

Beyond their canonical roles in autophagy, several ARPs significantly influence radiotherapy response through distinct molecular mechanisms. Genetic silencing of activating transcription factor 4 (ATF4), a key regulator of autophagy-related gene expression, significantly enhances radiosensitivity in TNBC. This effect is mediated primarily through the disruption of glutathione (GSH) biosynthesis, leading to catastrophic accumulation of ROS and subsequent induction of oxidative stress-mediated apoptosis [94,95]. Pharmacological inhibition of heat shock protein 70 (HSP70), despite its known role in promoting autophagy, enhances radiation efficacy by inducing G2/M cell cycle arrest, amplifying radiation-induced ROS generation, and impairing DNA damage repair [96]. These findings uncover a previously underappreciated paradigm in which ARPs modulate radiation response via both canonical (autophagy-dependent) and non-canonical (autophagy-independent) mechanisms. Notably, their non-autophagic roles constitute complementary therapeutic vulnerabilities for targeted radiosensitization.

### 4.3. Immunotherapy

Immunotherapy has fundamentally transformed the therapeutic landscape for TNBC, with immune checkpoint inhibitors (ICIs) emerging as a breakthrough modality, particularly in advanced and metastatic disease. These agents exert their antitumor effects by disrupting tumor-mediated immunosuppressive mechanisms, thereby reinvigorating cytotoxic T cell function and restoring endogenous antitumor immunity [97,98,99]. Despite these clinical advances, therapeutic benefits remain restricted to a subset of patients. Mounting evidence now implicates ARPs as key determinants of immunotherapy response, orchestrating critical tumor-immune crosstalk through dynamic remodeling of the tumor immune microenvironment, regulation of immune checkpoint expression patterns, and functional reprogramming of immune cell populations.

Autophagy significantly influences immunotherapy outcomes by modulating distinct cell death pathways. For instance, the CMA-associated protein HSPA8 directly governs ferroptosis sensitivity by facilitating the lysosomal degradation of glutathione peroxidase 4 (GPX4). Notably, HSPA8 depletion leads to GPX4 stabilization, conferring tumor cell resistance to ferroptosis and ultimately compromising PD-1 blockade efficacy [100]. Moreover, intact autophagic flux is essential for sustaining an immunologically active tumor microenvironment. In autophagy-impaired TNBC models, defective cargo clearance results in pathological p62/SQSTM1 accumulation, which in turn stabilizes the immunosuppressive matricellular protein Tenascin-C (TNC). This TNC upregulation establishes a physical and immunological barrier that limits CD8^+^ T cell infiltration and promotes immune evasion. Importantly, therapeutic intervention with TNC-neutralizing antibodies reverses this immunosuppressive phenotype and restores sensitivity to PD-1 blockade therapy [65].

ARPs further regulate immunotherapy responses through microenvironmental signaling pathways. HMGB1, a damage-associated molecular pattern (DAMP) released during autophagy, triggers proinflammatory cytokine secretion that paradoxically fosters an immunosuppressive tumor microenvironment. Pharmacological inhibition of HMGB1 not only attenuates this cytokine storm but also enhances T cell-mediated antitumor immunity and synergizes with PD-1 blockade therapy [101].

Additionally, emerging evidence reveals that mitophagy serves as a key regulatory mechanism controlling PD-L1 protein turnover. The mitochondrial AAA^+^ ATPase ATAD3A has been identified as a critical negative regulator of this process, where it actively suppresses PINK1-mediated mitophagy and consequently inhibits PD-L1 degradation. Intriguingly, PTX chemotherapy was found to significantly upregulate ATAD3A expression, resulting in PD-L1 stabilization and potentially contributing to acquired resistance to immune checkpoint blockade therapy. Most notably, targeted inhibition of ATAD3A was shown to rescue PINK1-dependent mitophagic flux, effectively promoting PD-L1 clearance and significantly enhancing the antitumor efficacy of combined PTX and immune checkpoint inhibitor treatment [102,103].

In summary, autophagy-related proteins orchestrate TNBC immunotherapy responses through a multifaceted regulatory network, influencing immune checkpoint expression, tumor cell death pathways, and the immunosuppressive microenvironment. Strategic modulation of autophagy pathways in combination with immunotherapy represents a promising therapeutic paradigm to enhance response rates and achieve sustained clinical benefits in TNBC treatment.

## 5. ARPs as Biomarkers in TNBC

ARPs exhibit temporally regulated, stage-dependent expression patterns across the TNBC disease continuum, establishing their dual role as both pathogenic drivers and clinically exploitable biomarkers. These molecular signatures provide enhanced early detection through distinct proteomic fingerprints, refined molecular classification beyond conventional histopathology, prognostic stratification via strong associations with metastatic progression and survival outcomes, and predictive capacity for chemotherapy, radiation, and immunotherapy response. This multidimensional clinical utility positions ARPs as transformative tools for precision oncology, enabling data-driven therapeutic decision-making from diagnosis through metastatic disease management (Table 1).

### 5.1. Diagnostic Value of ARPs in TNBC

ARPs display unique subtype-specific expression patterns in TNBC, serving as powerful molecular discriminators that reliably distinguish TNBC from both normal breast tissue and other breast cancer subtypes. A particularly notable example is ATG7, which shows marked specific downregulation in TNBC relative to other subtypes of breast cancer and normal breast tissue [40]. This unique expression pattern likely reflects the characteristic metabolic reprogramming and altered autophagy dependence of TNBC, positioning ATG7 as a promising diagnostic biomarker for TNBC identification.

### 5.2. Prognostic Significance of ARPs in TNBC

ARPs exhibit significant associations with clinical outcomes in TNBC, functioning as highly informative prognostic biomarkers that reliably predict metastatic progression and disease severity. For instance, eEF2K overexpression associates with reduced overall survival, increased lymph node/distant metastasis, and larger tumor size [59,134]. ATF4/HSPA8 overexpression correlates with decreased survival rates [52,107]. Low DAB2IP expression predicts poor overall prognosis, whereas high DAB2IP expression in chemotherapy-treated patients associates with improved survival, highlighting its dual prognostic and predictive value [58].

### 5.3. Predictive Value of ARPs for Therapy in TNBC

Multi-gene signatures incorporating ARPs have become indispensable for predicting treatment response and survival outcomes in TNBC, enabling personalized, risk-stratified therapeutic approaches. Among these, two clinically relevant signatures demonstrate particular prognostic value. An eight-gene autophagy signature, including ATF4, TGFBR1, SMAD4, and PIK3CA, correlates significantly with relapse-free survival (RFS), serving as a tool for prognosis and risk stratification [107]. Another eight-gene autophagy signature containing ATG7, ATG12, HSPA8, and WIPI1 predicts patient survival risk, with ATG12 amplification and WIPI1 downregulation significantly associated with clinical stage, providing insights into disease severity and treatment response [112].

Although the potential of ARPs as biomarkers for TNBC is increasingly recognized, the specificity of individual markers is limited. There is an urgent need to develop multi-marker combined models based on large-scale, multi-center data.

## 6. Therapeutic Strategies Targeting ARPs in TNBC

ARPs play pivotal roles in TNBC progression and therapeutic resistance, establishing them as attractive but challenging therapeutic targets. Unlike global autophagy modulation, effective therapeutic approaches require precise regulation of autophagic flux or selective interference with the tumor-promoting functions of specific ARPs. Strategies must be carefully tailored to both the tumor microenvironment and treatment regimen. Although preclinical investigations have revealed several promising agents targeting distinct phases of autophagy, critical obstacles, particularly the context-dependent dichotomous roles of ARPs and the marked heterogeneity inherent to TNBC, remain to be addressed before successful clinical implementation can be achieved.

At the autophagy initiation stage, the serine/threonine kinase ULK1 serves as the master regulatory switch governing autophagosome formation. Emerging preclinical evidence highlights several promising ULK1-targeting therapeutic strategies. The small-molecule inhibitor SBI-0206965 selectively inhibits ULK1 and synergizes with mTOR inhibitors to promote ULK1 degradation and enhance tumor cell apoptosis, outperforming chloroquine (CQ) in efficacy [135]. In vitro, SBI-0206965 sensitizes DOX-resistant TNBC cells to high-dose DOX, suppressing their malignant phenotype [136]. However, its poor oral bioavailability poses a clinical limitation [137].

More recently, SBP-7455, a dual ULK1/2 inhibitor with higher target affinity than SBI-0206965, has been shown to block starvation-induced autophagy in TNBC and synergize with PARP inhibitors like olaparib [138]. Another inhibitor, MRT67307, exhibits dual activity by suppressing both ULK1/2-mediated autophagy and TBK1-dependent interferon signaling, thereby modulating immune and inflammatory pathways [139]. Notably, the ULK1 inhibitor XSI-14 targets cancer stem cells by blocking autophagosome formation, while its derivative A4 demonstrates potent antitumor effects and favorable safety in vivo when combined with sorafenib [140,141].

The Beclin-1-VPS34 complex serves as a key therapeutic target during autophagosome nucleation. SAR405, a selective PIK3C3/VPS34 inhibitor, disrupts lysosomal function and suppresses starvation-induced autophagy. Notably, its combination with the mTOR inhibitor everolimus demonstrates synergistic antiproliferative effects in renal carcinoma cells [142]. In TNBC models, SAR405 exhibits dual functionality by both enhancing NF-κB signaling and activating innate immune cells, suggesting immunomodulatory antitumor potential [68].

Spautin-1 represents another promising intervention strategy through its inhibition of USP10/13, leading to VPS34 complex degradation and autophagy suppression. Recent findings reveal an additional mechanism whereby Spautin-1 promotes TOMM complex-mediated PINK1-PRKN-dependent mitophagy [143]. This compound shows particular therapeutic relevance in TNBC, where its combination with PTX effectively overcomes chemoresistance by impairing mitochondrial quality control and inducing cell death in PTX-resistant populations [144]. The well-characterized autophagy inhibitor 3-methyladenine (3-MA) remains a valuable research tool, functioning through specific inhibition of VPS34 lipid kinase activity. By preventing PI3P generation, 3-MA effectively blocks the initial stages of autophagosome formation [34,145,146].

At the degradation stage, CQ and hydroxychloroquine (HCQ) represent the most clinically advanced autophagy modulators. These lysosomotropic agents function by alkalizing lysosomal pH and inhibiting hydrolytic enzymes, effectively blocking autophagosome–lysosome fusion and subsequent cargo degradation [75,147,148]. In TNBC, CQ demonstrates multifaceted therapeutic effects, including suppression of radiation-induced metastasis, selective targeting of CSCs, reducing their population and metastatic potential, and synergistic enhancement of carboplatin efficacy [93,149]. The combination of CQ with doxorubicin demonstrates dual therapeutic benefits, including significant reduction in breast CSC tumorigenicity and potentiation of chemotherapy-induced cytotoxicity [150]. This synergy may be attributed to CQ’s ability to block autophagy-mediated survival pathways in treatment-resistant cell populations. More strikingly, triple-combination therapy incorporating CQ with PI3K/AKT inhibitors and PTX achieves effective reversal of protective autophagy triggered by PI3K/AKT pathway inhibition and marked enhancement of tumor cell apoptosis, and potentially overcomes multiple drug resistance mechanisms [151]. CQ combined with isorhamnetin promotes mitochondrial damage and apoptosis [152]. Recent advances in drug delivery have further optimized CQ’s therapeutic profile. Nanocarrier-based formulations improve tumor targeting while minimizing off-target toxicity [150,153]. Beyond these clinical agents, bafilomycin A1 represents another potent autophagy inhibitor that blocks late-stage autophagy through disruption of lysosomal function. Notably, it has shown promise in reversing cisplatin resistance in squamous cell carcinoma models [154,155].

## 7. Future Perspectives

The formidable clinical challenges presented by TNBC underscore the critical need for novel therapeutic strategies. Emerging evidence positions ARPs as a promising new frontier in TNBC treatment, offering multifaceted opportunities for intervention. This comprehensive review elucidates the pivotal roles of these proteins in driving TNBC pathogenesis and modulating treatment responses, establishing them as master regulators of disease progression, key determinants of therapeutic resistance, and attractive targets for clinical translation.

These proteins collectively drive tumor malignancy across multiple levels. At the cellular level, they influence tumor growth by regulating the cell cycle and apoptotic balance. At the tissue level, they promote disease dissemination through modulating migration capacity and epithelial–mesenchymal transition. At the microenvironmental level, they reshape immune states and thereby affect interactions between the tumor and the host. This multifaceted regulation establishes autophagy-related proteins as pivotal nodes within the complex TNBC network.

In treatment contexts, ARPs exhibit dual roles. On one hand, they may enhance cell survival and contribute to therapy resistance. On the other hand, targeted interventions, such as lysosome-directed chloroquine drugs, can effectively reverse resistance and improve therapeutic outcomes. Additionally, their potential as biomarkers offers new avenues for precision diagnosis and treatment of TNBC.

Although existing studies have elucidated some functions and mechanisms of autophagy-related proteins, the precise regulatory networks across different molecular subtypes, disease stages, and treatment settings remain incompletely understood, and their clinical utility requires further validation. Future research must deepen understanding of the dual roles of autophagy, alongside innovative approaches to overcome current challenges in drug development and biomarker validation. With continued advances in basic and translational research, precise stratification and individualized intervention for TNBC are expected to gradually become achievable, providing new breakthroughs for this difficult-to-treat breast cancer.

## Figures and Tables

**Table 1:** Expression status and Functions of ARPs in TNBC.

Protein	Change	Phenotype	Mechanism	Reference
ALDH1A3	Upregulation	Promotes adhesion and migration, facilitating the initiation of early metastasis	Enhances fibrinolytic activity through transcriptional regulation mediated by retinoic acid (ATRA), promoting extracellular matrix degradation	[104,105]
		Promotes tumor growth and progression, increasing the proportion of cancer stem cells	Induces the expression of lncRNA NRAD1	[106]
ATF4	Upregulation	Radiation resistance	Activates transcription of GSH synthesis genes, inhibiting ROS accumulation	[94,95]
		Promotes proliferation and invasion	Regulates through the TGFβ/SMAD2/3/4 and PI3K/mTORC2 pathways	[107]
ATG2B	Downregulation	Reduces cancer stem cell proportion and malignant tumor phenotype	Regulates by enhancing autophagy	[57]
ATG3	Downregulation	Induces cell death, inhibits tumor stem cell traits and migratory ability	Activates autophagy to promote apoptosis	[108]
ATG5	Upregulation	Maintains cell survival, proliferation, migration, and invasion capabilities	Activates autophagy to degrade pro-apoptotic protein PMAIP1	[95,109]
		Radiation resistance	Activates autophagy, alleviates DNA damage	[92]
ATG7	Downregulation	Inhibits proliferation, migration, EMT	Downregulates expression of key EMT transcription factors and mesenchymal markers	[40,110]
		Enhances chemotherapy-induced apoptosis	Activates autophagy and inhibits aerobic glycolysis	[110]
ATG9A	Upregulation	Promotes cell proliferation and invasion	Unknown	[111]
ATG12	Upregulation	Promotes tumor stemness and malignant progression	Regulates via enhanced autophagy	[112]
ATG13	Upregulation	Promotes cell proliferation, migration, invasion, and autophagy activation	Via circEGFR → TFEB → ATG13/ULK1 positive feedback loop, enhancing autophagy and thereby promoting malignant phenotype	[11]
ATG14	Unknown	Promotes cell survival	Regulates via enhanced autophagy	[113]
ULK1	Upregulation	Promotes cell proliferation, migration, and invasion	Via circEGFR → TFEB → ATG13/ULK1 positive feedback loop, enhancing autophagy and thereby promoting malignant phenotype	[11]
AURKA	Upregulation	Promotes cell proliferation, metastasis, and stemness	Regulates transcription factor SOX8, activating downstream target genes	[59]
		Promotes immune evasion	Through activation of MYC transcription, mediating PD-L1 upregulation	[114]
		Reduces radiosensitivity	By upregulating GPX4, inhibiting ferroptosis, and weakening radiotherapy-induced ROS effects	[115,116]
BAG3	Upregulation	Enhances chemotherapy resistance and malignant phenotype	Stabilizes anti-apoptotic BCL-2 family proteins, promotes protective autophagy, inhibits apoptosis, and upregulates EMT transcription factors	[77]
BDNF	Upregulation	Promotes colonization and growth of brain metastases	Activates the TrkB receptor on the cell surface	[117]
BECLIN 1	Upregulation	Promotes cell proliferation, migration, and invasion	Induces G0/G1 cell cycle arrest and promotes EMT	[33,34]
BNIP1	Downregulation	Inhibit cell proliferation and enhance autophagic cell death	Activates autophagy	[118]
BNIP3	Upregulation	Supports cell survival, proliferation, and metastasis	Acts synergistically with GPCPD1-mediated mitophagy pathway	[119]
CAMK2A	Upregulation	Promotes tumor cell invasion and metastasis	Promotes STAT3 phosphorylation at Tyr705, upregulates downstream target genes, driving EMT and extracellular matrix degradation	[120]
CAMKK2	Upregulation	Promotes cell migration and invasion	Activates the PDE1A-PKG1-VASP axis, promoting actin cytoskeleton assembly	[121]
CCL2	Upregulation	Induces immune evasion and reduces sensitivity to immunotherapy	Recruits MDSCs to remodel the immune microenvironment	[122]
		Promotes EMT, cell invasion, and stemness	Activates AKT kinase, phosphorylating β-catenin at Ser552 to translocate into the nucleus, upregulating EMT and stem cell markers	[123]
		Promotes lung metastasis	Directly inhibiting CCR2 signaling and recruiting inflammation	[124]
DAB2IP	Downregulation	Suppresses stemness and chemoresistance	Inhibiting nuclear translocation of β-catenin	[58]
DNAJB1	Upregulation	Promotes docetaxel resistance and reduces cell cycle arrest and apoptosis	Regulating mutp53/TAp63	[125]
DNAJB9	Downregulation	Inhibits EMT and metastasis	Stabilizing FBXO45 and promoting ubiquitination of ZEB1	[126]
DYNLT1	Upregulation	Promotes proliferation, colony formation, migration, and invasion	Enhancing mitochondrial metabolism via DYNLT1-Parkin-VDAC1	[127]
EEF2K	Upregulation	Promotes survival, proliferation, invasion, migration, and metastasis	Binding and phosphorylating AURKA at S391 to enhance stability and kinase activity, upregulating SOX8 expression	[59,128]
		Mediates chemoresistance	Activating DNA damage repair pathways	[83]
		Regulates immune evasion	Phosphorylating GSK3β to inhibit its activity and stabilizing PD-L1 expression	[59,128]
FOXO3	Downregulation	Promotes apoptosis	Activating downstream targets PINK1 and Parkin to facilitate mitophagy	[129]
FUNDC1	Upregulation	Promotes proliferation, colony formation, invasion, and metastasis	Mediating hypoxia-induced mitophagy	[130]
HSP90	Upregulation	Promotes proliferation, invasion, EMT, angiogenesis, and immune evasion	Sustaining EGFR/ERK signaling and regulating immunosuppressive molecule expression	[131]
STX4	Upregulation	Promotes migration, invasion, and distant metastasis	Binding Munc18c and forming functional complexes with other SNARE proteins to enhance invadopodia function	[132]
NBR1	Upregulation	Promotes metastatic potential	Stabilizing and activating transcription factor p63, enhancing expression of downstream targets such as CK5 and CK14	[53]
HMGB1	Upregulation	Promotes immune evasion	Inducing immune tolerance and recruiting immunosuppressive cells via the RAGE pathway	[101]
		Promotes chemoresistance	Activating autophagy and mediating drug efflux through ABCG2	[82]
HSPA8	Upregulation	Promotes proliferation and inhibits apoptosis	Degrading VAV1 via CMA, suppressing ERK pathway activation	[52]
		Regulates immunotherapy response	Inhibiting ferroptosis by stabilizing GPX4	[100]
WIPI2	Unknown	Promotes chemoresistance	Activating autophagy	[133]

## Abbreviations

ALDH1A3： aldehyde dehydrogenase 1 family member A3; AMPK, AMP-activated protein kinase; ARPs, autophagy-related proteins; ATAD3A, ATPase family AAA domain-containing 3A; ATF4, activating transcription factor 4; ATG12, autophagy related 12; ATG13, autophagy related 13; ATG14, autophagy related 14; ATG2B, autophagy related 2B; ATG3, autophagy related 3; ATG5, autophagy related 5; ATG7, autophagy related 7; ATG9A, autophagy related 9A; AURKA, aurora kinase A; BAG3, BAG cochaperone 3; BDNF, brain-derived neurotrophic factor; BECN1, beclin 1; BNIP1, BCL2 interacting protein 1; BNIP3, BCL2 interacting protein 3; CAMK2A, calcium/calmodulin-dependent protein kinase II alpha; CAMKK2, calcium/calmodulin-dependent protein kinase kinase 2; CCL2, C-C motif chemokine ligand 2; CMA, chaperone-mediated autophagy; CSCs, cancer stem cells; DAB2IP, DAB2 interacting protein; DNAJB1, DnaJ heat shock protein family member B1; DNAJB9, DnaJ heat shock protein family member B9; DOX, doxorubicin; DYNLT1, dynein light chain Tctex-type 1; EDEM1, ER degradation-enhancing alpha-mannosidase-like protein 1; EEF2K, eukaryotic elongation factor 2 kinase; EMT, epithelial–mesenchymal transition; ERK, extracellular signal-regulated kinase; FIP200, family interacting protein of 200 kDa; FOXO1, forkhead box O1; FOXO3, forkhead box O3; FUNDC1, FUN14 domain-containing 1; HECTD3, HECT domain E3 ubiquitin protein ligase 3; HMGB1, high-mobility group box 1; HSP90, heat shock protein 90; HSPA8, heat shock protein family A member 8; ITCH, itchy E3 ubiquitin protein ligase; mTOR, mechanistic target of rapamycin; NBR1, Next to BRCA1 gene 1 protein; PI3K, phosphatidylinositol 3-kinase; PINK1, PTEN-induced kinase 1; PRKN, parkin RBR E3 ubiquitin protein ligase; PTEN, phosphatase and tensin homolog; PTX, paclitaxel; ROS, reactive oxygen species; SNARE, soluble NSF attachment protein receptor; SQSTM1/p62, sequestosome 1; STX4, syntaxin 4; TFEB, transcription factor EB; TNBC, triple-negative breast cancer; UBQLN1, ubiquilin 1; ULK1, unc-51-like autophagy-activating kinase 1; VAMP3, vesicle-associated membrane protein 3; WIPI2, WD repeat domain phosphoinositide interacting 2.
